# Whistleblowing as an anti-corruption strategy in health and pharmaceutical organizations in low- and middle-income countries: a scoping review

**DOI:** 10.1080/16549716.2022.2140494

**Published:** 2022-11-10

**Authors:** Taryn Vian, Brianna Agnew, Keith McInnes

**Affiliations:** a Department of Health Professions, School of Nursing and Health Professions, University of San Francisco, San Francisco, CA, USA; b WHO Collaborating Centre for Governance, Accountability and Transparency in the Pharmaceutical Sector, Leslie Dan Faculty of Pharmacy, University of Toronto, Toronto, Canada; c Center for Healthcare Organization and Implementation Research (CHOIR), VA Bedford Healthcare System, Bedford, MA, USA; d Department of Health Law, Policy & Management, Boston University School of Public Health, Boston, MA, USA

**Keywords:** Whistleblowing, complaints, health systems, raising concerns, speaking up

## Abstract

**Background:**

Whistleblowing can bring suspected wrongdoing to the attention of someone who is in the position to rectify the problem. Whistleblowing research can help improve effectiveness of anti-corruption efforts in the health sector.

**Objective:**

The objective of this scoping review is to understand the extent and type of evidence on whistleblowing as an anti-corruption strategy in health and pharmaceutical organisations in low- and middle-income countries (LMICs).

**Methods:**

This scoping review searched the PubMed, Scopus, and EMBASE databases from 2005 to 2020, limited to English language. We also searched websites of multilateral agencies or international non-governmental organisations for policy documents, guidance and reports. Titles and abstracts were screened to remove those where the focus was not on health, pharmaceuticals, whistleblowing, or LMIC context. Articles focused on research misconduct were excluded. Full-text articles were assessed for eligibility on these same criteria. Included sources were analysed thematically, based on five categories including definitions and models; evidence of reporting frequency; factors influencing whistleblowing; cultural context; and outcomes.

**Results:**

The review found 22 sources including reports, policies, and guidance documents (12, 55%), news articles (4, 18%), policy analyses/reviews (3, 14%), commentaries (2, 9%), and empirical studies (1, 5%). Most sources described whistleblowing policy and system components such as how whistleblowing is defined, who can report, and how confidentiality is assured. Few articles documented types and frequencies of corruption identified through whistleblowing or factors associated with whistleblowing. Several studies mentioned cultural norms as a potential limitation to whistleblowing effectiveness. About one-third of the sources described fear of retaliation and noted the need to strengthen protection for whistleblowers.

**Conclusion:**

Research on whistleblowing is scarce in health and pharmaceutical organisations in LMICs. Documentation of policies, factors associated with whistleblowing, and whistleblowing outcomes is needed and could help countries to mainstream whistleblowing as a sectoral anti-corruption strategy.

## Background

Corrupt practices in health organisations include bribery, misappropriation of medicines, trading in influence, abuse of functions, false billing, and other illicit enrichment schemes [1]. Corruption and fraud in health and pharmaceutical organisations have enormous financial and social costs [2,3]. Fraud and abuse are estimated to cost $58.5 billion to $83.9 billion annually in the U.S. alone [4], and an estimated 7.3% of total health expenditures is lost to health care fraud and abuse worldwide each year, according to figures from the Centre for Counter Fraud Studies at the University of Portsmouth and the accounting firm PKF in the UK [2]. Abuse of power can corrupt the selection, procurement, distribution, and prescribing of medicines [5]. Researchers have documented the indirect health impact of corruption including increased infant, child, and maternal mortality, cancer deaths, motor vehicle crash deaths, earthquake-related fatalities, anxiety, poor general health, and antibiotic resistance [3,6]. Corruption in food and nutrition organisations can also adversely affect health [7]. While corruption is a problem in all countries, the impact of corruption on health is often more severe in low-income countries due to lower economic performance and quality of governance [8,9].

Whistleblowing, the act of raising concerns about suspected or observed wrongdoing to others in a position to effect action, is part of a national integrity system to prevent corruption [10,11]. Reporting by employees in the private sector accounts for 40% of fraud cases detected and is the single most important strategy for detecting occupational fraud committed by employees or managers [12,13]. Whistleblowing mechanisms are especially important during health crises such as the COVID-19 pandemic, when embezzlement of healthcare resources can lead to unsafe working conditions and poor clinical care [14–16]. In addition to deterring corruption, whistleblowing can reveal systemic weaknesses that allow wrongdoing. Organisations can use this information to strengthen health systems.

Often organisations understand the term whistleblowing to apply only to current or former employees [17,18]. Yet, patients and suppliers may have sufficient knowledge about wrongdoing to blow the whistle [19,20]. These external whistleblowers are sometimes called ‘reporters’ to distinguish them from whistleblowers on the inside [21]. Whistleblowing channels may be seen as a continuum – first reporting within the management structure, followed by external reporting if internal reporting is not effective [22].

A scoping review is an approach to rapidly map concepts related to a research topic [23]. The use of a scoping review is appropriate for this study, given that the types of evidence to address and inform the practice of whistleblowing in this context are not precisely known [24].

A preliminary search of PubMed, the Cochrane Library, and JBI Evidence Synthesis was conducted in September 2021 to find current systematic or scoping reviews related to whistleblowing, health, and corruption. This search found four reviews.

Kang conducted a systematic review of whistleblowing in the public sector to examine methods and theoretical models [25]. The review included 71 peer-reviewed publications and dissertations in public administration, political science, and business. Several studies included low- and middle-income countries (LMICs) such as China, Hungary, Serbia and Peru, and 8% of studies examined whistleblowing and corruption. None of the studies considered the health sector. Most empirical studies applied human resources, ethics, and psychology theories to assess individual, organisational, and situational factors associated with whistleblowing. Kang concluded that more research is needed on the frequency of whistleblowing, impact of leadership and training, effectiveness of legal protection systems, and consequences of whistleblowing [25].

Mugellini, Bella, Colagrossi, Isenring and Killas conducted a systematic review on public sector reforms and corruption [26]. The review included randomised controlled trials of interventions to curb corruption in any sector. The studies were conducted in high-, middle-, and low-income countries and examined control and deterrence interventions (e.g. reporting, audit, and transparency), and cultural and organisation interventions (e.g. codes of ethics, and training). Only two studies considered the health sector, and neither of these looked at whistleblowing. Five studies examined whistleblowing interventions outside the health sector, including guaranteeing immunity to whistleblowers. Researchers thought this intervention would increase whistleblowing and discourage bribes; however, evidence did not support their hypotheses. The authors recommend that anti-corruption interventions be grounded in economic theory (control and deterrence interventions) but also consider moral and cultural factors such as the political climate and individuals’ self-control or desire for power [26].

Blenkinsopp, Snowden, Mannion, Powell, Davies and Millar examined empirical studies of whistleblowing related to patient safety and quality of care [20]. The researchers found that most studies were carried out in Australia, U.K. and the U.S. (66% of studies reviewed), and few studies were conducted in LMICs. The researchers summarised evidence of factors influencing whistleblowing, including individual and role characteristics of whistleblowers, organisational culture and climate, and organisational leadership and management. They also described evidence on the consequences of whistleblowing. The authors observed that studies of healthcare whistleblowing are published in a wide variety of journals, suggesting that studies may not be building on prior theory and knowledge. They observed a dearth of research on the organisational response to whistleblowing and the impact of whistleblowing (e.g. what portion of healthcare whistleblowing results in retaliation versus positive responses).

Kelly & Jones conducted a narrative review focused on whistleblowing literature relevant to care of older people in Wales (domiciliary care organisations, hospitals, nurses, and doctors) [27]. They included opinion pieces, theoretical articles, and empirical literature from business, management, and healthcare journals. Kelly & Jones observed that there is no universally accepted definition of whistleblowing or theoretical framework guiding the practice in healthcare. Their review found no clear pattern on characteristics of whistleblowers in terms of sex or age, but they noted whistleblowers had better job performance, were more highly educated, scored higher on tests of moral reasoning, and held higher level positions than non-whistleblowers. Nurses tended to blow the whistle more than doctors. Their review found that organisational climate and clinical leadership influenced whether people chose to raise concerns or not. Climate and leadership also affected whether concerns were handled well or whistleblowers experienced retaliation [27].

None of the reviews included empirical evidence of whistleblowing involving people external to the organisation such as patients, nor did they consider how the ecosystem for whistleblowing may differ in LMICs. Neither of the two reviews that focused on health [20,27] reported findings related to whistleblowing on fraud or corruption. This suggests a need for a review of literature specifically focused on whistleblowing in healthcare, pharmaceutical, and other health-related organisations a) reporting concerns about corruption and fraud; b) including whistleblowing by patients or other knowledgeable reporters in addition to employees; c) in LMIC context; and d) considering many types of sources, not just empirical research.

The objective of this scoping review is to understand the extent and focus of the literature on whistleblowing as an anti-corruption strategy in health and pharmaceutical organisations in LMICs. The findings could help further our knowledge of how whistleblowing systems might be adapted for LMICs, and relevant factors influencing effectiveness in controlling corruption. This can help to inform the direction of future research, policy, and practice.

## Review question

The scoping review question is ‘In the context of LMIC health and pharmaceutical organizations, how is whistleblowing understood and what evidence do we have about whistleblowing as an anti-corruption strategy?’

Sub-questions include:

1. What definitions, models, and systems for whistleblowing have been used?
2. What evidence exists about the types and frequency of corruption reported through whistleblowing?
3. What do we know about the factors that influence whistleblowing?
4. How does country or cultural context affect whistleblowing?
5. What is known about the outcomes of whistleblowing, positive and negative?

## Methods

The scoping review was designed in accordance with the JBI methodology for scoping reviews [28,29]. Because this review did not involve human subjects research, ethical approval was not required. The protocol for the study was registered in the Open Science Framework database (https://osf.io/2w3g8) [30].

### Eligibility Criteria

Eligibility criteria for scoping reviews generally specify participants, concept, and context [31]. We focused on organisations, conceptualised as a body of people with a particular purpose. This included national government agencies (such as the ministry of health or medicines regulatory authority), multilateral organisations (including United Nations agencies), private companies, and non-governmental organisations. For studies including participants, we included articles mentioning internal whistleblowers, such as current or former employees, and external reporters such as patients and others who may have knowledge of or suspect wrongdoing, but are not employed by the organisation.

Concepts important to the search included whistleblowing and corruption. As mentioned earlier, our definition of whistleblowing is the act of raising concerns about suspected or observed wrongdoing to others in a position to effect action. We included articles that considered both corruption and safety or quality issues, but excluded articles that focused exclusively on whistleblowing for safety or quality concerns and did not mention corruption or fraud. Finally, we excluded articles related to whistleblowing and research-related fraud. While concerns such as plagiarism, data falsification, and data fabrication in health and pharmaceutical-related research are important, we are interested in corruption more directly affecting health system service delivery, health workforce, health information systems, medicines, financing, and leadership [32].

Concerning context, we included articles with a focus on LMICs, or both LMICs and high-income countries. We excluded articles focused on high-income countries exclusively. We suspect that the antecedents to whistleblowing and the policies and processes for handling complaints may have to be adapted in the context of LMICs due to cultural, economic, political and other considerations. We wanted to know what studies have already been done to consider these intertwining issues.

We were mainly interested in health- and pharmaceutical-related organisations, though we also included keywords for food, nutrition, water and sanitation (related areas). We excluded sources where the purpose was not related to health goals.

### Types of sources

The scoping review considered any type of study design. We included non-research sources such as commentaries, agency reports, and guidance documents. We included news articles related to whistleblowing systems but excluded news articles following corruption cases where whistleblowing was not the main focus.

### Search strategy

We searched PubMed, Scopus and EMBASE databases. An initial search of PubMed was undertaken to identify articles on the topic. The text words contained in the titles and abstracts were used to develop a full search strategy. The search strategy was then adapted for each included database and/or information source. The reference lists of included sources were searched for additional articles. We have searched the archives of the journals *Health Policy and Planning*, *International Journal of Health Policy and Management*, *Public Administration and Development*, and the *Third World Quarterly*.

Studies published in English language from 2005 forward were included. We chose 2005 because this is when the U.N. Convention Against Corruption (UNCAC) entered into force. This global treaty legally binds the 140 signatory countries to consider laws to protect people who report corruption-related offences from retaliation (Article 33). The ratification of UNCAC created impetus for work by member states and international organisations to support whistleblowing [33].

Sources of unpublished studies included the websites of the European Anti-Fraud Office; European Healthcare Fraud and Corruption Network; Gavi, the Vaccine Alliance; The Global Fund to Fight AIDS, TB, and Malaria; Group of States Against Corruption; the National Whistleblower Center (a U.S. non-governmental organisation); the Organisation for Economic Co-operation and Development; Transparency International; United Nations Development Programme; United Nations Office on Drugs and Crime; and the World Health Organization (WHO). Supplementary File 1 shows our initial keyword search in PubMed.

### Source selection

Identified citations were uploaded into *EndNote 20 (Clarivate Analytics, PA, USA)* and duplicates removed. We then conducted a pilot review of the screening process, with two reviewers (TV and BA) looking at 20 sources to see if the reviewers were interpreting the criteria in similar ways. Following the pilot test and discussion to reach a common understanding, the two reviewers independently screened all titles and abstracts against the inclusion criteria (Supplementary File 2a).

Sources were retrieved in full and their citation details saved. TV and BA independently assessed the full text of selected citations against the inclusion criteria. Reasons for exclusion of sources not meeting inclusion criteria were recorded. Disagreements between the reviewers were resolved through discussion. Following best practice, the results of the search and the study inclusion process are reported in the findings [29].

### Data extraction and analysis

Data were extracted from sources by the two reviewers using a worksheet (Supplementary File 2b). The data extracted included details about the citation, participants, concept, context, study type, and key findings relevant to the five review sub-questions, i.e. 1) definitions, models, and systems for whistleblowing; 2) types and frequency of corruption revealed through whistleblowing; 3) factors associated with whistleblowing; 4) country/cultural context and whistleblowing; and 5) impact or consequences of whistleblowing. Disagreements were resolved through discussion.

All three authors independently reviewed the extracted data and created notes about key findings. For example, the notes for an article with findings relevant to sub-question #1, would include the source’s definition of whistleblowing, describe the contextual setting and legislative or regulatory basis for the definition, and explain other components of the policy or system, such as the stated purpose, types of whistleblowers covered, and reporting methods. Notes for an article with findings related to sub-question #3 might describe in more detail the specific factors considered to influence the decision to blow the whistle, and the strength of evidence for the association with whistleblowing. The research team then discussed the notes together, and the results were organised into a synthesis outline with narrative summary under main conceptual categories. A PRISMA extension for Scoping Reviews (PRISMA-ScR) checklist was completed to guide reporting (Supplementary File 3).

## Findings

**Figure 1.**
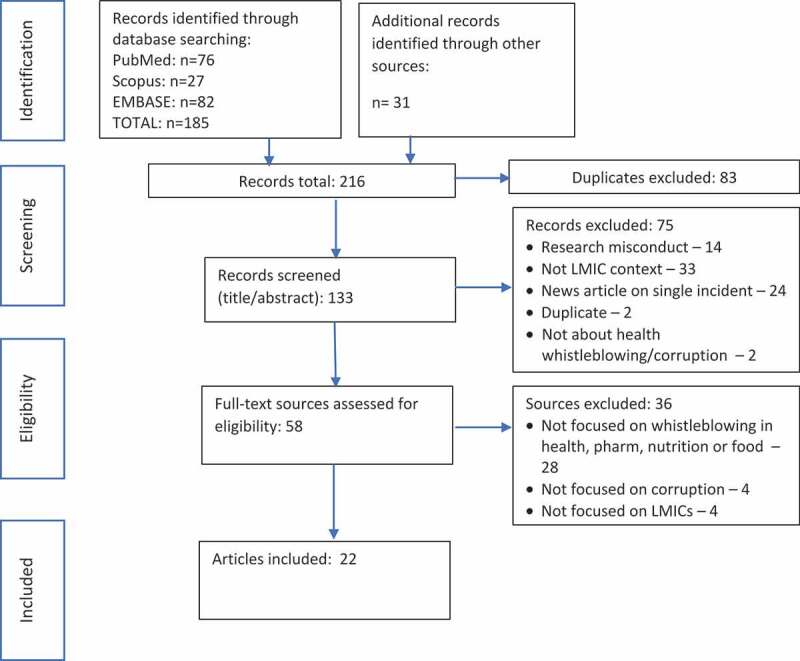
Article selection process .

Our search resulted in 216 records. After removing 83 duplicates, we reviewed the title and abstract of the remaining 133 records and excluded 75 sources that did not meet inclusion criteria (see Figure 1). Full texts were obtained for the remaining 58 sources and were assessed for eligibility. Thirty-six articles were excluded at this stage, including 28 (78%) that did not contain information on whistleblowing in the organisations of interest; 4 (11%) that were not concerned with corruption; and 4 (11%) that were not focused on the LMIC context (see Supplementary File 4). Ultimately, 22 sources were retained for this review, including organisational reports, policies & procedures, and guidance documents (12, 55%), news articles related to whistleblowing systems (4, 18%), policy analyses and reviews (3, 14%), commentaries (2, 9%), and empirical studies (1, 5%). Most sources (13, 59%) included information relevant to the health organisations; three sources (14%) considered pharmaceutical whistleblowing; two (9%) examined whistleblowing related to food supply; and four (16%) considered a mix of these areas.

Sources from the non-peer-reviewed literature included policies and procedures for whistleblowing promulgated by the European Union, WHO, the Global Fund to Fight AIDS, Tuberculosis, and Malaria, and Gavi, the Vaccine Alliance. While the policy of the European Union is not specific to the health sector, the goal of the policy mentions protection of health.

No significant changes to the protocol were made when implementing the scoping review. Below we describe the importance of context to understanding evidence on whistleblowing policies and systems, and present our results organised by review sub-question.

### Context for whistleblowing

High profile corruption cases can be impetus to adopting whistleblowing systems. The Global Fund mentions publicised fraud cases, monetary and reputational losses as rationale for a whistleblowing policy [34]. Policy in the European Union was set in context of global scandals affecting all countries such as the Panama papers and Cambridge Analytica [35]. Some articles mentioned how whistleblowing may be the only way of gaining knowledge about high level corruption involving powerful interests [36,37]. Whistleblowing systems were also seen as a source for identifying systems-level malfeasance and areas needing improvement [36,38]. Some agencies mentioned the urgency of whistleblowing because of risks with COVID vaccine distribution and other supplies related to infectious disease control [39,40], along with the need to protect human health, animal health, and public health [35,41].

### Sub-question 1: definitions, models, and systems for whistleblowing

The first study sub-question asked what definitions, models, and systems for whistleblowing were described in the sources identified for the scoping review. Twenty out of 22 articles (91%) were coded as having relevant information.

Definitions of whistleblowing mentioned reporting to expose wrongdoing, serious misconduct, or serious breaches of rules, guidance, or policy (see Table 1). The purpose of whistleblowing policy was often described as increasing accountability and transparency, minimising or managing risks, fulfiling duties, and minimising financial, health, and reputational effects of the wrongdoing.

**Table 1.** Definition and purpose of whistleblowing.

Organisation	Definition of Whistleblowing or whistleblower	Purpose
Global Fund [34,42]	“A pathway to report and expose corruption within an organisation [involving] open disclosure about significant wrongdoing made by a concerned citizen totally or predominantly motivated by notions of public interest.” [34] “To blow the whistle is to alert a third party that a person or entity has done, or is doing, something wrong. So, literally, “whistle-blowing” means that a party, in good faith, conveys or transmits a concern, allegation or information indicating that a prohibited practice is occurring or has occurred in the Global Fund or in a Global Fund-financed operation.” [42]	To improve accountability and transparency to ensure organisational credibility and effectiveness of funding. To manage risks effectively and prevent, detect, and stand up to corruption. To maintain trust. To fulfill the Global Fund’s “duty … to act as a responsible custodian or trustee of funds entrusted to it, by protecting the interests and assets of all its stakeholders—donor countries, recipient countries, or diverse beneficiaries alike.”
United Nations Office on Drugs and Crime [15]	According to article 8 of the UN Convention Against Corruption, states must establish “measures and systems to facilitate the reporting by public officials of acts of corruption to appropriate authorities.”	To detect serious instances of wrongdoing and address them as soon as possible in order to mitigate negative impact on health, finances, and the country’s reputation.
European Union [15,35,43]	Whistleblowers are “reporting persons working in the private or public sector who acquired information on breaches in a work-related context.”	An essential “upstream” component of law enforcement, providing information that leads to effective detection, investigation, and prosecution. It can help prevent and detect procurement-related fraud and corruption, and unfair or illicit manufacturing, import, or distribution of unsafe products.
Gavi [44]	“‘Whistleblower’ means an individual who reports suspected incidents of breaches of laws, rules or regulations in Gavi’s activities or of serious misconduct or serious infringement of Gavi’s rules, policies or guidelines, or any action that is or could be harmful to the mission or reputation of Gavi (each, a ‘Wrongdoing’).”	“To encourage reporting of suspected wrongdoing that may threaten the operations or governance of Gavi … without fear of retaliatory action, and to enable Gavi to effectively address such wrongdoing, manage risks and uphold standards of good governance.”
WHO [45]	“Whistleblowers [are defined] as individuals who report suspected wrongdoing that implies a significant risk to WHO, i.e. harmful to its interests, reputation, operations or governance.”	“To encourage the reporting of suspected wrongdoing when the wrongdoing implies significant corporate risk (i.e. harmful to the interests, reputation, operations, or governance of WHO) without fear of retaliatory action in order to enable WHO to take early action.”
Food Crime Management Systems [37]	Whistleblowing is “disclosure by organizational members of illegal, immoral, or illegitimate practices that are executed under the control of their employers, to persons or organizations that may be able to effect action as a result of that disclosure.”	Whistleblowing is seen as a management control strategy whose purpose is to deter potential food safety issues, recalls, and profit losses. To help businesses to identify and manage risks better.

#### Who can report

Most often, the policies and guidance specified who can be considered a whistleblower, including staff, former staff, and others who would know about suspected or actual wrongdoing in the work context [42,46]. ‘Other reporters’ might be people interviewing for a job, suppliers or vendors, and volunteers [35,42]. A few sources mentioned ‘consumers’ as potential whistleblowers. For example, a website created by a citizen group in India was advertised as a place where consumers and employees could raise issues [38]. Only one source directly mentioned patients as whistleblowers [47].

#### Rewards and incentives

Our review found few references to whistleblowing policies structured to reward whistleblowers monetarily. Multilaterals and global funding mechanisms did not have reward policies [42,44,45], possibly because they see whistleblowing as a staff obligation grounded in duty [42,45]. However, financial incentive programmes were mentioned as operating in the U.S., India, China, and the Republic of Korea [22,46,48].

A whistleblowing system was implemented in India in 2010 by the Ministry of Health and Social Welfare and the Central Drugs Standard Control Organisation to combat substandard and falsified medical products. It included a reward scheme equivalent to 20% of the value of the seized consignment of fake medicines, to a maximum value of Rs 25 Lakh ($54,750 USD). Rewards for individual government officials were capped at Rs 5 Lakh ($10,950) per consignment and Rs 30 Lakh ($65,700) maximum during the officer’s career [46,48].

#### Anonymity and confidentiality

Policies varied in whether they allow anonymous complaints. All policies we reviewed had provisions for protecting confidentiality of reporting individuals, but where a channel does allow anonymous reporting, it is not possible to protect the reporter from retaliation (if someone later suspects or discovers the reporter). In India, the whistleblowing portal created for medical professionals and the public was only for anonymous complaints. Those managing the portal didn’t want to know who reported on whom, because the purpose of the portal was systems improvement, not detection for prosecution [38]. A pharmaceutical sector whistleblowing reward programme in India protected confidentiality, but they needed the name of the reporter to provide the reward so reporting was not anonymous [46].

Global Fund whistleblowing can be anonymous or confidential [42]. Gavi and WHO policies say they will protect the confidentiality of the reporter and shield them from retaliation; however, anonymous reporters cannot be protected from retaliation [44,45]. Moy [22] describes how a whistleblowing programme in Russia requires the witness to go public with the report. Policy in Bulgaria also does not allow anonymous reporting [41].

#### Whistleblower protections

Whistleblowers often face retaliation [36,47,49]. Whistleblowing policies include a definition and examples of retaliation [15,44,45]. For example, the UN defines retaliation as ‘any direct or indirect detrimental action that adversely affects the employment or working conditions of an individual, where such action has been recommended, threatened or taken for the purpose of punishing, intimidating or injuring an individual’ as a result of the individual having reported misconduct [15].

The United Nations Office on Drugs and Crime whistleblowing guidance acknowledges ‘personnel will not come forward if they are unsure that protective measures will be put in place to minimize the risk they are taking.’ Protections from retaliation should extend to witnesses of the wrongdoing, colleagues and family of the whistleblower, and those who helped facilitate reporting [15]. Possible measures include [15,41]:

- Guaranteeing confidentiality; punishing officials who reveal the identity;
- Reassigning or reinstating the whistleblower when appropriate;
- Putting in place mechanisms to report, investigate and sanction retaliation;
- Providing support to whistleblowers (counselling, advice, financial support).

WHO provides access to an ‘ethics advice’ line and a process for reporting retaliation and reviewing retaliation claims [45]. The European Union Directive on Whistleblowing states that whistleblowers should be protected if they have reasonable grounds to believe allegations are true [43]. Because whistleblowers may have violated loyalty or confidentiality to share this information, they could lose their jobs. Regular citizens do not face that imbalance [43]. Low socioeconomic status of workers may make them especially susceptible to retaliation for reporting fraud [22]. India passed a Whistleblower Protection Act that became law in 2014. The law has provisions to punish public officials who break the confidentiality of the whistleblower with up to three years in prison and a fine of Rs 50,000 ($805 USD). However, the law does not provide protection for private workplace retaliation and provides no implementation details [50].

#### Internal versus external whistleblowing

Systems can encourage whistleblowers to disclose internally to someone in direct chain of command, or externally to regulatory officers, inspectors, auditors, police, Members of Parliament, citizen groups, or media. One example of an external whistleblowing system is Transparency International’s worldwide network of Advocacy and Legal Aid Centres currently operating in more than 60 countries (https://www.transparency.org/en/alacs). The centres collectively received more than 1,800 reports from victims of wrongdoing or witnesses to corruption related to COVID-19 in countries such as the Democratic Republic of Congo, Ireland, Kenya, Nigeria, Russia, the United Kingdom, Venezuela, and Zimbabwe [51,52].

Yet, the definition of internal versus external is not always clear. For example, Gavi’s Audit and Investigation unit operates several whistleblowing channels, accepting reports via a web-based portal, by mail and email, and through voice-messaging. It then has a three-stage process to assess reports to determine if it requires further investigation. Some might consider this external reporting, since the group receiving the reports is not in the direct line of command; however, it is still within the Gavi organisation. We found no evidence of empirical studies involving health or pharmaceutical organisations to document attitudes and perceptions about internal versus external whistleblowing.

### Sub-question 2: types and frequency of corruption reported through whistleblowing

The second sub-question of our review was to determine what evidence exists about the types and frequency of corruption reported through whistleblowing. Five out of 22 articles (23%) were coded as having relevant information.

The analysis shows that whistleblowing at multilateral organisations is increasing over time. We also found concerns about retaliation may be suppressing whistleblowing in other types of organisations, such as government agencies.

In 2015, the Global Fund OIG’s office launched a campaign with resources for anti-corruption, transparency, and accountability [53]. Researchers believe the campaign may have helped increase reporting over time [34]. From 2013 to 2020, Gavi received about three whistleblower reports per year [39]. Reports substantially increased to 67 in 2021, but a large number were categorised as ‘non-substantive, misdirected … or reflect anti-vaxxer sentiment.’ In other words, the issue raised did not have a significant impact on the organisation’s activities or was not about wrongdoing. After investigation, 16 were found to have merit. These included 14 related to vaccine equity (i.e. complaints about deviations from agreed upon vaccine priorities) and two related to core Gavi activities. The equity complaints were mostly at the start of the COVID vaccine delivery process, and such complaints are now slowing, possibly because supply became less limited [39].

**Figure 2.**
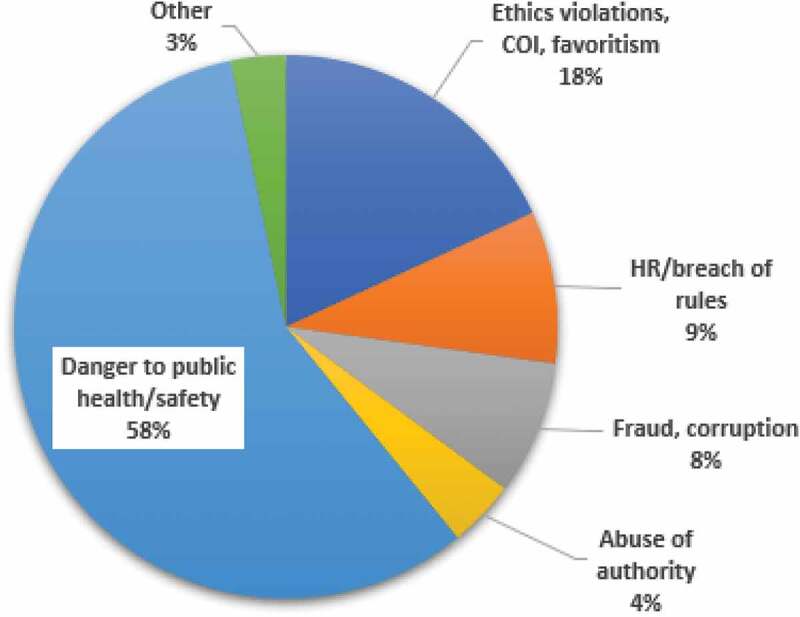
WHO integrity hotline complaints, Jan-Nov, 2020.

Between Jan and Nov 2020, WHO received 276 reports from staff and the general public through its Integrity Hotline (Figure 2). Fifty (18.1%) were on breaches of code of ethics and professional conduct, conflict of interest, discrimination and favoritism; 25 (9.0%) were related to human resource issues and breaches of staff regulations/rules; 22 (8.0%) were about suspected fraud, corruption or bribery; 11 (4.0%) were about abuse of authority and harassment; and 159 (57.6%) sounded alarm about substantial danger to public health and personal safety, mainly related to the COVID-19 pandemic. No information was available on whether investigations were completed or the outcomes of such investigations. WHO also reported a significant increase in people seeking ethics advice, continuing a pattern from 2019. In 2020, they received 400 individual requests, possibly due to awareness-raising activities and COVID-19 [40].

Fourteen percent of the anti-fraud experts surveyed by Milata [49] reported the emergence of whistleblowers related to COVID-19 fraud and corruption, including reports related to procurement fraud and medical bribes to access COVID testing and treatment.

Though the previous examples show an increase in whistleblowing reports to multilateral organisations, reporting at the national level could be suppressed where people fear retaliation. Moy reported that only three in 10 individuals in South Africa felt safe blowing the whistle, despite the official Protected Disclosures Act of 2000, which protects public and private employees who disclose information of unlawful or corrupt conduct by their employers or fellow employees [22]. A newspaper report in 2021 described how a whistleblower in the Gauteng Province Department of Health in South Africa was killed in a shooting, possibly because she was a witness in the investigation of COVID-related fraud [54].

### Sub-question 3: factors influencing whistleblowing

The third review sub-question was to assess what is known about factors that influence whistleblowing. Seven of the 22 sources (32%) described facilitators and impediments to reporting wrongdoing.

#### Facilitators

We did not identify empirical studies of factors affecting whistleblowing in the health sector in LMICs. However, four factors were mentioned as being possible facilitators: financial rewards for whistleblowing or financial support after experiencing retaliation, appeal to duty, assurance of confidentiality, and having multiple channels for reporting. In addition, patients who cannot afford to pay informally may blow the whistle on health staff who obstruct their access to care.

The prospect of a financial reward was seen as an important motivating factor in India [46]. In contrast, the Global Fund relies on employees’ perception of professional responsibility. The Global Fund describes this duty in their whistleblowing policy [42]:

This whistle-blowing policy springs from the duty of the Global Fund to act as a responsible custodian or trustee of funds entrusted to it, by protecting the interests and assets of all its stakeholders – donor countries, recipient countries, or diverse beneficiaries alike.

European Partners Against Corruption and European Contact-point Network Against Corruption mentioned that offering financial and psychological assistance to whistleblowers through an independent fund might reduce fear of retaliation [41]. The promise of confidentiality was seen as important to encourage reporting in India [46]. Policies often include many provisions to protect confidentiality throughout the investigation process.

Having multiple channels for reporting can also be a facilitating factor. Some people are more comfortable reporting in person, others prefer to report in writing (submit by post, physical complaint box or an online platform), or would like to report orally by telephone or voice message [43]. General research on whistleblowing suggests that whistleblowing practices may differ for men versus women; women may lack means and resources to report, may be particularly influenced by social norms, and have greater fear of retaliation [55]. This suggests that gender sensitivity is needed in designing reporting channels.

Finally, patients and their families may be more inclined to blow the whistle if they believe there is no other way to access care. In Morocco, a father who blew the whistle had been asked for a speed bribe to get needed care for his child [47]. He could not pay, and saw blowing the whistle to the police as the only way to get care.

#### Impediments

The most frequently mentioned impediments to whistleblowing were fear of retaliation and perceived lack of protection (discussed further under sub-question 5). A second, related factor is lack of trust. Some people may fear reporting because they suspect that the higher-level officials, to whom they are reporting, may also be corrupt [47]. The European Union Directive on Whistleblowing states that ‘Lack of confidence in the effectiveness of reporting is one of the main factors discouraging potential whistleblowers’ [43].

Other factors include lack of knowledge that people are legally entitled to assistance from authorities if they are asked for a bribe [47], and fear on the part of outside companies that they will lose revenue if they report knowledge of corruption [48].

### Sub-question 4: how does country or cultural context affect whistleblowing?

The fourth review sub-question was to consider evidence on how country or cultural context affects whistleblowing. Only three sources (14%) included relevant information.

Chatterjee claimed that India’s endemic corruption makes people less willing to blow the whistle [50]. The author noted a number of violent attacks on Indian whistleblowers, and extreme cases of retaliation such as two medical officers from Uttar Pradesh who were murdered after having reported healthcare corruption. Additionally, medical professionals have been harassed by officials and fired from jobs [50]. This situation may be similar in the Middle East and North Africa region, where Transparency International has noted that whistleblowing is ‘almost completely unheard of’ and no countries have adequate protections [56].

Moy observes that cultural context can be an impediment to whistleblowing. In some cultures, the whistleblower is perceived negatively and stereotyped as a disgruntled employee or disloyal worker [22]. Although Moy does not mention specific cultures, Soon & Manning note that in China, personal relationships, fear of retaliation, and media coverage may discourage whistleblowing [37]. *Guanxi* refers to existing or potential informal relationships based on reciprocal obligation in the workplace and socially [57]. If a person has a good personal relationship, or *guanxi*, with a work colleague, they may be reluctant to blow the whistle and risk damaging that relationship or not fulfiling the obligation for loyalty and trust that it assumes. Drawing media coverage may also be feared because it could impact *guanxi* [37,58].

### Sub-question 5: what are the impacts and consequences of whistleblowing?

The final sub-question for the scoping review was to understand what is known about the outcomes of whistleblowing for whistleblowers and for the organisation itself. Eight out of 22 sources (36%) included information relevant to this review question.

#### Negative consequences

Articles described how organisations or individuals retaliate against whistleblowers. Whistleblowers risk career and livelihood, and may suffer financial, health, reputational and personal consequences [22,35]. Retaliation includes workplace harassment, job loss, workplace restrictions, and reduction of responsibilities [15,36,47,50]. Health workers who reported misconduct were subjected to severe official reprisals including demotion, reprimand, and psychiatric referral [37]. They were threatened, treated as a ‘traitor’, pressured to resign, or stalled in career progression [37,48]. Some faced false criminal conduct charges [36]. In extreme cases, whistleblowers have suffered physical harm or death [50].

Actions to suppress whistleblowers were documented through a survey of anti-fraud professionals from 58 countries [49]. Researchers reported that in 46% of countries represented, whistleblowers had been suppressed.

#### Positive impacts

We found little documentation of how whistleblowing led to prosecution or system changes. The European Union’s 2019 directive on the protection of persons who report breaches of Union law describes how whistleblowing could enhance food safety and prevention of disease transmission through the detection of fraud and corruption and subsequent enforcement and prosecution [43]; yet, we could not find reports to document actual outcomes.

Introduction of a whistleblowing mechanism and *I Speak Out Now!* campaign in Malawi led to increased arrests, fines, and prosecutions [34], though no further information was available. In Morocco, a whistleblower complaint about under-the-table payments resulted in police launching a sting operation. An undercover agent offered an informal payment to the nurse who had been identified by the whistleblower. After accepting the informal payment, the nurse was arrested, prosecuted, and received a sentence of two months in prison [47]. The patient was given the care they needed. Yet, while this may be a good deterrence strategy in the short term, the reporting was not used to identify the drivers of this type of corruption or identify sustainable solutions. Longer-term, sustainable anti-corruption strategies should use whistleblower information to address root causes of the informal payment practice, such as low wages and drug shortages [59]. None of the sources reviewed provided evidence of how organisations may have used information gained from whistleblowers to determine the drivers of corruption, or to strengthen health systems.

## Discussion

This scoping review was designed to explore the extent and type of evidence in the literature on whistleblowing as an anti-corruption strategy in health and pharmaceutical organisations in LMICs. We found that most of the literature is descriptive or normative, including policies, guidance, reports and commentary. We found no theoretical articles or scholarly critiques of whistleblowing for anti-corruption in the areas of interest. Our study revealed little evidence of data such as surveys, court cases, case studies, or administrative data being collected and used to study whistleblowing or develop policy and procedures. There is little research on how whistleblowing complaints in the health sector are being used to strengthen health systems. This suggests a broken feedback loop and disconnect between the national integrity system and organisational change.

International agencies working in the health arena have promulgated whistleblowing policies and guidance that can help inform country-level health and pharmaceutical sector policy. These policies and guidance documents include definitions and justifications, details on who is covered by the policy, channels for whistleblowing and procedures for investigating claims, provisions for confidentiality, and protection from retaliation. Yet, we did not find empirical evidence to justify policy effectiveness. An important area of future research should be to evaluate whistleblowing policies and programmes, to determine if this is a worthwhile investment at the sectoral level.

Our findings support the ideas raised in previous systematic reviews, i.e. that current research provides little insight on the way whistleblowing is approached in different healthcare systems, and the importance of studying how to provide support for whistleblowers within specific organisational contexts [20]. Similar to findings by Kelly and Jones [27], we found that few sources focused on the complex environment and interactions affecting whistleblowing at the personal and organisational level. It is important to understand how these factors intertwine to influence in the decision to blow the whistle, and how they may affect organisational responsiveness [27]. Theories may be useful in guiding this work of understanding motivational factors [13,60–62].

Case study research may also show how factors in the environment (such as leadership style, organisational culture and values, design features of the whistleblowing system such as modalities of reporting) interact with personal characteristics (gender, tenure, organisational role, personality) to affect whistleblowing frequency, and how the organisation uses the information to address corruption risks and strengthen health systems. For example, researchers in Brazil found that women reported wrongdoing less often than men, and experienced more retaliation [63]. Although public service motivation increased intent to blow the whistle (and was stronger in women), the experience of past retaliation suppressed intent to blow the whistle in the future. The researchers hypothesise that the relationship between gender and whistleblowing may be related to the low power-status position of women in organisations. Thus, in cultures where gender inequalities are less important, and legal foundations for whistleblowing are stronger, gender effects may be minimised [63].

Our scoping review has several implications for research. First, empirical work is needed to collect data from whistleblowing reports, court cases, or surveys to analyse types and frequencies of whistleblowing, and the personal, situational, and organisational factors facilitating whistleblowing for anti-corruption in the health and pharmaceutical organisations. Surveys and qualitative work to explore attitudes and perceptions of those involved (potential whistleblowers, managers, etc.) may provide data that can help guide and adapt policies. Empirical work can help us to evaluate factors that contribute to the decision to blow the whistle, and moderating factors that influence the strength or direction of the relationship. Programme evaluations can help us to determine if policies are effective, and what impacts result for the person blowing the whistle, and the organisation as a whole.

Secondly, building on empirical findings, more conceptual work is needed to develop theory on whistleblowing for anti-corruption in the health and pharmaceutical sector in LMICs. Researchers should document and critique whistleblowing models and strategies in health and pharmaceutical organisations. Strategies may vary across countries due to differing social norms, political environment, and structure of the health and pharmaceutical sectors. This information can be correlated with empirical data on whistleblowing perceptions, impact and consequences to develop theory and guide effective policies and protection mechanisms. Researchers should involve civil society actors to gain their perspectives.

Third, a critically important area for research is effective interventions to protect whistleblowers. The lowest income countries have most risk of corruption, yet they are most challenged by lack of resources to protect whistleblowers. Possibilities of protection for whistleblowers managed through third parties could be considered, building on examples of contracting out of government procurement services as an anti-corruption strategy [64].

Finally, whistleblowing systems may require culture change to overcome negative stereotypes of whistleblowers as traitors who cannot be trusted, and to build a culture of improvement. Further study is needed to understand the impact of cultural beliefs and norms on willingness to report, and how to combat negative perceptions of whistleblowing. This can draw on research designs from other sectors [65,66].

There are several limitations to our study. First, given timing and resource constraints, we chose to include three academic databases for this review along with grey literature. Yet, as others have noted, whistleblowing literature is not well developed in the health sector and may appear in other disciplines not captured. Searching more databases could identify additional sources. Secondly, the exclusion of articles in the review that considered research-related fraud could mean that important experiences of applying whistleblowing in the pharmaceutical sector (where there are likely many examples due to the inclusion of whistleblowing hotlines as part of corporate compliance schemes) were not captured, limiting the findings. Investigation into whistleblowing and research-related fraud in LMICs could be a topic for further research. Third, scoping reviews are designed to consider the extent, range, and nature of literature in the topic area and not to assess the quality of research. Thus, it is possible that some findings from these sources lack adequate evidence due to problems with the quality of methods used. Fourth, we limited the search to English language only. This may have excluded relevant articles published in other languages. Finally, we did not examine whistleblowing policies in health systems at the national or sub-national level in LMICs, or do specific outreach to find policies of international organisations that might not be available online. This might be a useful exercise for future work in this field.

## Conclusion

Corruption in the health sector is a serious and growing problem, especially affecting those who are most disadvantaged [8]. Whistleblowing can reduce the frequency and cost of corruption by allowing organisations to identify potential wrongdoing and address problems at an early stage. Yet, in order to work, whistleblowing systems must be carefully designed and adapted to context, with protections in place for whistleblowers. This review found that limited research has been conducted on whistleblowing in health, pharmaceutical, and related organisations focusing on anti-corruption and fraud in LMICs. Many avenues for further research could be productive and help inform policy and practice. This includes surveys, case studies, and other types of empirical research to examine the factors associated with the decision to report and the impact of whistleblowing on the organisation and the person blowing the whistle. Documentation of existing policies and sharing of experiences in policy implementation at all stages could help countries seeking to mainstream whistleblowing as a sector-specific anti-corruption strategy.

## Supplementary Material

Supplemental Material

## Paper context

Little is known about how whistleblowing is understood and used to detect corruption in health and pharmaceutical organisations in LMICs. This scoping review documented components of whistleblowing policies implemented by international organisations, and factors that may affect motivation for whistleblowing. We need further research to understand how whistleblowing systems operate in LMIC contexts, factors that enhance the effectiveness of such systems, and outcomes experienced by organisations and individuals involved.

## References

[1] United Nations Office on Drugs and Crime. United nations convention against corruption. New York: United Nations; 2004.

[2] Jones B, Jing A. Prevention not cure in tackling health-care fraud. Bull World Health Organ. 2011 Dec 1;89:858–14. 22271939 10.2471/BLT.11.021211 PMC3260891

[3] Vian T. Anti-corruption, transparency and accountability in health: concepts, frameworks, and approaches. Glob Health Action. 2020;13:1694744. 32194010 10.1080/16549716.2019.1694744 PMC7170369

[4] Shrank WH, Rogstad TL, Parekh N. Waste in the US health care system: estimated costs and potential for savings. JAMA. 2019 Oct 15;322:1501–1509. 31589283 10.1001/jama.2019.13978

[5] Kohler J, Dimancesco D. The risk of corruption in public pharmaceutical procurement: how anti-corruption, transparency and accountability measures may reduce this risk. Glob Health Action. 2020;13:1694745. 32194011 10.1080/16549716.2019.1694745 PMC7170361

[6] National Academies of Sciences Engineering and Medicine. The critical health impacts of corruption. In: National academies of sciences engineering and medicine, editor. Crossing the global quality chasm: Improving health care worldwide. Washington (DC): The National Academies Press; 2018. p. 203–225. 30605296

[7] Uchendu FN, Abolarin TO. Corrupt practices negatively influenced food security and live expectancy in developing countries. Pan Afr Med J. 2015;20:110. 26090058 10.11604/pamj.2015.20.110.5311 PMC4458312

[8] Achim M, Văidean V, Borlea S. Corruption and health outcomes within an economic and cultural framework. Eur J Health Econ. 2020;21:195–207. 31587123 10.1007/s10198-019-01120-8

[9] Holmberg S, Rothstein B. Dying of corruption. Health Econ Policy Law. 2011;6:529–547. 20809992 10.1017/S174413311000023X

[10] Organization for Economic Cooperation and Development [OECD]. OECD Public Integrity Handbook. Paris: OECD Publishing; 2020.

[11] UNODC. State of integrity: a guide on conducting corruption risk assessments in public organizations. Vienna: United Nations; 2020.

[12] Samuels J, Pope K. Are organizations hindering employee whistleblowing?. J Accountancy. 2014 [cited 2022 Aug 5]. https://www.journalofaccountancy.com/issues/2014/dec/employee-whistleblowers-corporate-fraud.html

[13] Owusu G, Bekoe R, Anokye F, Okoe F. Whistleblowing intentions of accounting students: an application of the theory of planned behavior. J Financ Crime. 2020;27:477–492.

[14] Armitage R, Nellums LB. Whistleblowing and patient safety during COVID-19. EClinicalMedicine. 2020 Jul;24:100425. 32766540 10.1016/j.eclinm.2020.100425 PMC7311911

[15] United Nations Office on Drugs and Crime. Speak-up for health: guidelines to enable whistle-blower protection in the health-care sector. Vienna: UNODC; 2021. pp. 1–76.

[16] OECD. Policy measures to avoid corruption and bribery in the COVID-19 response and recovery. Paris: OECD Publishing; 2020.

[17] Pohjanoksa J, Stolt M, Suhonen R, Löyttyniemi E, Leino-Kilpi H. Whistle-blowing process in healthcare: from suspicion to action. Nurs Ethics. 2019;26:526–540. 28494645 10.1177/0969733017705005

[18] Greene A, L JK. Whistle-blowing as a form of advocacy: guidelines for the practitioner and organization. Soc Work. 2004;49:219–230. 15124962 10.1093/sw/49.2.219

[19] Corruption Watch. The whistleblower’s handbook. South Africa: Corruption Watch; 2020.

[20] Blenkinsopp J, Snowden N, Mannion R, Powell M, Davies H, Millar R, et al. Whistleblowing over patient safety and care quality: a review of the literature. J Health Organ Manag. 2019;33:737–756. 31625824 10.1108/JHOM-12-2018-0363

[21] United Nations Office on Drugs and Crime. The United Nations convention against corruption resource guide on good practices in the protection of reporting persons. United Nations. 2015;1–99. https://www.unodc.org/documents/corruption/Publications/2015/15-04741_Person_Guide_eBook.pdf

[22] Moy GG. The role of whistleblowers in protecting the safety and integrity of the food supply. NPJ Sci Food. 2018;2:8. 31304258 10.1038/s41538-018-0017-5 PMC6550153

[23] Arksey H, O’Malley L. Scoping studies: towards a methodological framework. Int J Soc Res Methodol. 2005;8:19–32.

[24] Munn Z, Peters MDJ, Stern C, Tufanaru C, McArthur A, Aromataris E. Systematic review or scoping review? Guidance for authors when choosing between a systematic or scoping review approach. BMC Med Res Methodol. 2018;18:143. 30453902 10.1186/s12874-018-0611-x PMC6245623

[25] Kang MM. Whistleblowing in the public sector: a systematic literature review. Rev Public Pers Adm. 2022;0734371X2210787. DOI:10.1177/0734371X221078784

[26] Mugellini G, Bella SD, Colagrossi M, Isenring GL, Killias M. Public sector reforms and their impact on the level of corruption: a systematic review. Campbell Syst Rev. 2021;17:e1173. 10.1002/cl2.1173 PMC8356278 37131927

[27] Kelly D, Jones A. When care is needed: the role of whistleblowing in promoting best standards from an individual and organizational perspective. Qual Ageing. 2013;14:180–191.

[28] Peters MDJ, Marnie C, Tricco AC, Pollock D, Munn Z, Alexander L, et al. Updated methodological guidance for the conduct of scoping reviews. JBI Evid Synth. 2020;18:2119–2126. DOI:10.11124/JBIES-20-00167 33038124

[29] Tricco AC, Lillie E, Zarin W, O’Brien KK, Colquhoun H, Levac D, et al. PRISMA extension for scoping reviews (PRISMA-ScR): checklist and explanation. Ann Intern Med. 2018;169:467–473. 30178033 10.7326/M18-0850

[30] Vian T, Agnew B, McInnes K. Whistleblowing as an anti-corruption strategy in health and pharmaceutical organizations: a scoping review protocol. Open Science Framework. 2021. DOI:10.17605/OSF.IO/2W3G8 PMC9661981 36356311

[31] Levac D, Colquhoun H, O’Brien KK. Scoping studies: advancing the methodology. Implement Sci. 2010;5:69. 20854677 10.1186/1748-5908-5-69 PMC2954944

[32] World Health Organization. Everybody’s business: strengthening health systems to improve health outcomes – WHO’s framework for action. Geneva: WHO; 2007.

[33] Transparency International. Whistleblower protection and the UN convention against corruption. Berlin: Transparency International; 2013.

[34] Chang Z, Rusu V, Kohler JC. The global fund: why anti-corruption, transparency and accountability matter [Article]. Glob Health. 2021;17. DOI:10.1186/s12992-021-00753-w PMC8449911 34537059

[35] European Commission. Frequently asked questions: whistleblower protection. Memo 18/3442. Brussels: European Commission; 2018. pp. 1–5.

[36] Reynolds L. Not up for discussion: applying lukes’ power model to the study of health system corruption comment on “we need to talk about corruption in health systems” [Note]. Int J Health Policy Manag. 2019;8:723–726. 31779300 10.15171/ijhpm.2019.75 PMC6885860

[37] Soon J, Manning L. Whistleblowing as a countermeasure strategy against food crime. Br Food J. 2017;119:2630–2652.

[38] Bagcchi S. Website to report corruption in medicine launches in India. BMJ. 2014 Dec 8;349:g7539. DOI:10.1136/bmj.g7539. 25487875

[39] Gavi. Report to the board from audit and investigations, 30 November - 2 December 2021. Geneva: Gavi; 2021.

[40] World Health Organization. Compliance, risk management and ethics: annual report. EBPBAC34/4. Geneva: WHO Programme, Budget, and Administration Committee of the Executive Board; 2021. pp. 1–6.

[41] European Partners against Corruption (EPAC). European contact-point network against corruption (EACN). Manual on preventing corruption and promoting integrity. Austria: EPAC/EACN; 2022. pp. 1–314.

[42] The Global Fund Office of the Inspector General. Whistle-blowing policy and procedures for the global fund to fight AIDS, tuberculosis and malaria. The Global Fund. 2019;1–7.

[43] European Union. Directive (EU) 2019/1937 of the European parliament and of the council of 23 October 2019 on the protection of persons who report breaches of Union law. Luxembourg: European Union; 2019. pp. 1–39.

[44] Gavi. Gavi alliance whistleblowing policy version 4.0. Geneva: Gavi; 2020.

[45] World Health Organization. WHO whistleblowing and protection against retaliation. Policy and procedures. Geneva: WHO Office of Compliance, Risk Management, and Ethics; 2015. pp. 1–14.

[46] Bhan A. Health ministry institutes reward scheme for whistle-blowers reporting spurious drugs [Note]. Natl Med J India. 2010;23:56. 20839601

[47] Zouanat S. Exposing corruption in Morocco’s hospitals [Internet]. Berlin: Transparency International; 2019. Available from: https://www.transparency.org/en/blog/exposing-corruption-in-moroccos-hospitals.

[48] Jayaraman K. Failure of Indian whistleblower scheme points to deeper woes. Nat Med. 2010 Apr;16:364. 10.1038/nm0410-364a 20376034

[49] Milata P. Fraud’s impact on healthcare during COVID-19. Global survey on fraud and corruption affecting healthcare systems during COVID-19 in April 2020. Berlin: Nemexis; 2020. pp. 1–66.

[50] Chatterjee P. Whistleblowing in India: what protections can doctors who raise concerns expect? BMJ. 2015;350:350. 10.1136/bmj.h763 25712178

[51] Transparency International. COVID-19 makes women more vulnerable to corruption[Internet]. Berlin: Transparency International; 2020. [cited 2022]. Available from: https://www.transparency.org/en/news/covid-19-makes-women-more-vulnerable-to-corruption

[52] Transparency International. How corruption is making people sick [Internet]. Berlin: Transparency International; 2020 [cited 2022]. Available from: https://www.transparency.org/en/news/how-corruption-is-making-people-sick

[53] The Global Fund Office of the Inspector General. Anti-fraud and corruption tool kit for implementers. The Global Fund. 2017. https://www.ispeakoutnow.org/resources-en/

[54] Flanagan J. Covid corruption whistleblower shot dead Times; 2021 25 Aug [cited 2022 21 Sep]. Available from: link.gale.com/apps/doc/A673126852/AONE?u=usfca_gleeson&sid=bookmark-AONE&xid=86ac1a84

[55] Zúñiga N. Gender sensitivity in corruption reporting and whistleblowing. U4 helpdesk answer. Norway: U4 Anti-Corruption Resource Centre. 2020;10:1–11.

[56] Transparency International. Speaking up safely: civil society guide to whistleblowing middle East and North Africa region; 2015. p. 1–41.

[57] Bedford O. The relation between guanxi and interpersonal trust in the workplace. Integr Psychol Behav Sci. 2022;56:385–404. 34618341 10.1007/s12124-021-09658-0

[58] Hwang D, Staley B, Chen Y, Lan J-S. Confucian culture and whistle-blowing by professional accountants: an exploratory study. Manag Audit J. 2008;23:504–526.

[59] Peiffer C, Armytage R, Marquette H, Gumisiriza P. Lessons from reducing bribery in Uganda’s health services. Dev Policy Rev. 2020;39:721–739.

[60] Anvari F, Wenzel M, Woodyatt L, Haslam S. The social psychology of whistleblowing: an integrated model. Organ Psychol Rev. 2019;9:41–67.

[61] Zakaria M, Azmawaty Abd Razak S, Yusoff M. The theory of planned behavior as a framework for whistle-blowing intentions. Rev Eur Stud. 2016;8:221–236.

[62] Cho Y, Song H. Determinants of whistleblowing within government agencies. Public Pers Manage. 2015;44:450–472.

[63] Tavares GM, Lima FV, Michener G. To blow the whistle in Brazil: the impact of gender and public service motivation. Regul Gov. 2021 June;2021:1–19.

[64] Crown Agents. Combating heart disease and child and adult cancer: crown agents in new partnership with Ukrainian ministry of health: Crown Agents; 2019 [cited June 29, 2022]. Available from: https://www.crownagents.com/news-item/combating-heart-disease-and-child-and-adult-cancer-crown-agents-in-new-partnership-with-ukrainian-ministry-of-health

[65] Cheng X, Karim K, Lin K. A cross-cultural comparison of whistleblowing perceptions. Int J Manag Decis Mak. 2015;14:15.

[66] Park H, Rehg M, Lee D. The influence of confusian ethics and collectivism on whistleblowing intentions: a study of South Korean public employees. J Bus Ethics. 2005;48:387–403.

