# Supplementary material for: Whistleblowing as an anti-corruption strategy in health and pharmaceutical organizations in low- and middle-income countries: a scoping review
Source: Glob Health Action. 2022 Nov 10;15(1):2140494. doi: 10.1080/16549716.2022.2140494 (PMC9661981; doi:10.1080/16549716.2022.2140494)
Supplement: Supplemental Material [file ZGHA_A_2140494_SM7660.docx]

### **Supplementary File 1: Search strategy for PubMed**

| Dimension | Keywords |
| --- | --- |
| Participants | Not applicable |
| Concept | **Terms related to whistleblowing:**  Whistleblow* (to capture “whistleblow” and “whistleblowing”)  Whistle-blow* (to capture “whistle-blow” and “whistle-blowing”)  Blow* the whistle (to capture “blow the whistle” and “blowing the whistle”)  ("Whistleblowing"[Mesh]) to use Medical Subject Heading (MeSH) search capability  **Terms related to corruption:**  Corruption  Bribe* (includes “bribe” and “bribery”)  Kickback  Embezzl* (captures embezzling, embezzlement, embezzle)  Steal  Theft  Patronage  ("Fraud"[Mesh]) to use MeSH search capability in PubMed. In other databases, Fraud will simply be a keyword for search. |
| Context | **Terms related to health and pharmaceutical sectors:**  Health  Healthcare  Primary care  Hospital*  Pharmaceutical*  Medicine*  Drug*  Food  Nutrition  Water  Sanitation  Hygiene |
| Limits | English language, 2005 to 2021 |

### **Supplementary File 2a: Study selection review form**

| **Concept** | **Description** | **Meets Criteria** |
| --- | --- | --- |
| **Inclusion Criteria** |  |  |
| Whistleblowing | Focus on whistleblowing or whistleblowers |  |
| Corruption | Focus corruption OR both corruption and safety/quality of care |  |
| **Exclusion Criteria** |  |  |
| Not research misconduct | Does not exclusively focus on research misconduct |  |
| **Context** |  |  |
| **Inclusion Criteria** |  |  |
| Geography | Focus on LMICs, OR both LMICs and high-income countries |  |
| Sector | Focus on health or pharmaceutical organizations or nutrition or water & sanitation |  |
| **Other** |  |  |
| **Inclusion Criteria** |  |  |
| Language | Written in English |  |
| Dates | Published between 2005 and 2021 |  |
| **Exclusion Criteria** |  |  |
| Source type | Not news articles |  |

###

### **Supplementary File 2b: Data extraction form**

| **ITEM** |  |
| --- | --- |
| **STUDY DETAILS** | |
| Author(s) |  |
| Title |  |
| Year |  |
| Journal |  |
| Vol/Issue |  |
| Pages/Article # |  |
| Study type (empirical, theoretical, policy or guidance, commentary/opinion, other) |  |
| Participants (employees, former employees, patients, family members, vendors, other, none) |  |
| # Participants (if relevant) |  |
| Year(s) of data collection |  |
| **CONCEPT** | |
| Whistleblowing for what (corruption alone, corruption & patient safety/quality, other) |  |
| Sector or organization (healthcare, pharmaceutical, nutrition, water and sanitation) |  |
| **CONTEXT** | |
| Country or countries |  |
| **FINDINGS** | |
| **Relevant to which research sub-question(s)**  #1 Definitions, models, or systems thinking about whistleblowing  #2 Evidence of types/frequency of observed or suspected wrongdoing reported through whistleblowing  #3 Motivational antecedents or situational/organizational facilitating or inhibiting factors affecting whistleblowing  #4 How country or cultural context affects whistleblowing  #5 Consequences of whistleblowing |  |

**Supplementary File 3: Preferred Reporting Items for Systematic reviews and Meta-Analyses extension for Scoping Reviews (****PRISMA-ScR) Checklist**

| **SECTION** | **ITEM** | **PRISMA-ScR CHECKLIST ITEM** | **REPORTED ON PAGE #** |
| --- | --- | --- | --- |
| **TITLE** | | | |
| Title | 1 | Identify the report as a scoping review. | 1,2, and 4 |
| **ABSTRACT** | | | |
| Structured summary | 2 | Provide a structured summary that includes (as applicable): background, objectives, eligibility criteria, sources of evidence, charting methods, results, and conclusions that relate to the review questions and objectives. | 2 (abstract) |
| **INTRODUCTION** | | | |
| Rationale | 3 | Describe the rationale for the review in the context of what is already known. Explain why the review questions/objectives lend themselves to a scoping review approach. | 4-5 |
| Objectives | 4 | Provide an explicit statement of the questions and objectives being addressed with reference to their key elements (e.g., population or participants, concepts, and context) or other relevant key elements used to conceptualize the review questions and/or objectives. | 6-7 |
| **METHODS** | | | |
| Protocol and registration | 5 | Indicate whether a review protocol exists; state if and where it can be accessed (e.g., a Web address); and if available, provide registration information, including the registration number. | 7 |
| Eligibility criteria | 6 | Specify characteristics of the sources of evidence used as eligibility criteria (e.g., years considered, language, and publication status), and provide a rationale. | 7-8 |
| Information sources* | 7 | Describe all information sources in the search (e.g., databases with dates of coverage and contact with authors to identify additional sources), as well as the date the most recent search was executed. | 8 |
| Search | 8 | Present the full electronic search strategy for at least 1 database, including any limits used, such that it could be repeated. | 9 and Supplementary File 1 |
| Selection of sources of evidence† | 9 | State the process for selecting sources of evidence (i.e., screening and eligibility) included in the scoping review. | 9-10 and Supplementary Files 2a and 4 |
| Data charting process‡ | 10 | Describe the methods of charting data from the included sources of evidence (e.g., calibrated forms or forms that have been tested by the team before their use, and whether data charting was done independently or in duplicate) and any processes for obtaining and confirming data from investigators. | 9-10 and Supplementary File 2b |
| Data items | 11 | List and define all variables for which data were sought and any assumptions and simplifications made. | Supplementary File 2a and 2b |
| Critical appraisal of individual sources of evidence§ | 12 | If done, provide a rationale for conducting a critical appraisal of included sources of evidence; describe the methods used and how this information was used in any data synthesis (if appropriate). | NA |
| Synthesis of results | 13 | Describe the methods of handling and summarizing the data that were charted. | 10 |
| **RESULTS** | | | |
| Selection of sources of evidence | 14 | Give numbers of sources of evidence screened, assessed for eligibility, and included in the review, with reasons for exclusions at each stage, ideally using a flow diagram. | 10-11 and Figure 1 |
| Characteristics of sources of evidence | 15 | For each source of evidence, present characteristics for which data were charted and provide the citations. | 10-11 |
| Critical appraisal within sources of evidence | 16 | If done, present data on critical appraisal of included sources of evidence (see item 12). | NA |
| Results of individual sources of evidence | 17 | For each included source of evidence, present the relevant data that were charted that relate to the review questions and objectives. | 12-20 |
| Synthesis of results | 18 | Summarize and/or present the charting results as they relate to the review questions and objectives. | 12-20 |
| **DISCUSSION** | | | |
| Summary of evidence | 19 | Summarize the main results (including an overview of concepts, themes, and types of evidence available), link to the review questions and objectives, and consider the relevance to key groups. | 21-22 |
| Limitations | 20 | Discuss the limitations of the scoping review process. | 22 |
| Conclusions | 21 | Provide a general interpretation of the results with respect to the review questions and objectives, as well as potential implications and/or next steps. | 23 |
| **FUNDING** | | | |
| Funding | 22 | Describe sources of funding for the included sources of evidence, as well as sources of funding for the scoping review. Describe the role of the funders of the scoping review. | 24 |

JBI = Joanna Briggs Institute; PRISMA-ScR = Preferred Reporting Items for Systematic reviews and Meta-Analyses extension for Scoping Reviews.

* Where *sources of evidence* (see second footnote) are compiled from, such as bibliographic databases, social media platforms, and Web sites.

† A more inclusive/heterogeneous term used to account for the different types of evidence or data sources (e.g., quantitative and/or qualitative research, expert opinion, and policy documents) that may be eligible in a scoping review as opposed to only studies. This is not to be confused with *information sources* (see first footnote).

‡ The frameworks by Arksey and O’Malley (6) and Levac and colleagues (7) and the JBI guidance (4, 5) refer to the process of data extraction in a scoping review as data charting*.*

§ The process of systematically examining research evidence to assess its validity, results, and relevance before using it to inform a decision. This term is used for items 12 and 19 instead of "risk of bias" (which is more applicable to systematic reviews of interventions) to include and acknowledge the various sources of evidence that may be used in a scoping review (e.g., quantitative and/or qualitative research, expert opinion, and policy document).

**Supplementary File 4: Whistleblowing Scoping Review References Excluded After Screening**

Reasons for Exclusion: NC=Not related to corruption; NL=Not low- or middle-income context; NWH=Not directly focused on whistleblowing and/or health-related

1. Achary, A., et al. (2019). "Role of whistle blowers in health care industry: An empirical study." Indian Journal of Public Health Research and Development 10(1): 142-146. [NC]
2. Ahmed, H. (2019) Building a whistleblowing culture in Palestine. Transparency International blog. <https://www.transparency.org/en/blog/building-a-whistleblowing-culture-in-palestine> [NWH]
3. ASJ Honduras. (2019). Corruption revealed in Honduran hospital. <https://www.transparency.org/en/blog/corruption-revealed-in-honduran-hospital> [NWH]
4. Blanchfield, L. (2012). United Nations reform: U.S. policy and international perspectives. Globalization: Trade Agreements, Global Health and United Nations Involvement**:** 117-149. [NWH]
5. Bouchard, M., et al. (2012). "Corruption in the health care sector: A barrier to access of orthopaedic care and medical devices in Uganda." BMC Int Health Hum Rights 12: 5. [NWH]
6. Braillon, A. (2010). "Whistleblowing: neither reward, nor protection." J Public Health Policy 31(2): 278-279 [NL]
7. Chapman, M., Hollingsworth, V., Aviram, A., and Rees, M. (2021). Smoke screen: BAT, bribes, and spies in the tobacco industry. Bureau of Investigative Journalism. <https://www.thebureauinvestigates.com/stories/2021-09-14/smoke-screen-british-american-tobacco-bribes-and-spies-in-the-tobacco-industry> [NWH]
8. Council of Europe. (2014). Protection of Whistleblowers. Recommendation CM/Rec(2014)7 adopted by the Committee of Ministers of the Council of Europe on 30 April 2014 and explanatory memorandum. <https://rm.coe.int/16807096c7> [NWH]
9. Eban, K. (2020). "Can we trust the quality of generic drugs?" Journal of Managed Care and Specialty Pharmacy 26(5): 589-591 [NWH]
10. European Union. (2018). Whistleblower protection fact sheet. [NWH]
11. Gee, J. (2009). "Mobilizing the honest majority to fight health-sector fraud." Bull World Health Organ 87(4): 254-255. [NWH]
12. IFC Consulting Services. (2020). Barriers and enablers of the reporting of intelligence regarding food crime. Final Report. Submitted to U.K. Food Standards Agency. <https://www.food.gov.uk/sites/default/files/media/document/food-crime-intelligence-reporting-barriers-and-enablers.pdf> [NL]
13. Kesselheim, A. S., et al. (2011). "Strategies and practices in off-label marketing of pharmaceuticals: a retrospective analysis of whistleblower complaints." PLoS Med **8**(4): e1000431. [NL]
14. Liu, C. (2014). “Guest Post: India’s Whistleblower Protection Act — An Important Step, But Not Enough.” Global Anti-Corruption Blog. [NWH]
15. Loncarek, K. (2008). "When you hear the whistle blow." Croatian medical journal **49**(4): 570-574. [NWH]
16. Ministry of Law and Justice, India (2014). The Whistleblowers Protection Act 2011. [NWH]
17. Mulinari, S. (2016). "Unhealthy marketing of pharmaceutical products: An international public health concern." Journal of Public Health Policy 37(2): 149-159. [NWH]
18. Nicholls, A. R., et al. (2021). "Snitches Get Stitches and End Up in Ditches: A Systematic Review of the Factors Associated with Whistleblowing Intentions." Front Psychol **12**: 631538. [NWH]
19. Novak, A., et al. (2020). "Anti-corruption policy under the conditions of overcoming the consequences of the coronavirus pandemic." Systematic Reviews in Pharmacy **11**(10): 911-916. [NWH]
20. OECD. (2011, Nov 25). Study on Whistleblower Protection Frameworks, Compendium of Best Practices and Guiding Principles for Legislation. Developed to support the G20 Anti-Corruption Action Plan Protection of Whistleblowers. Paris: OECD. <https://www.oecd.org/corruption/48972967.pdf> [NWH]
21. OECD. (2014). Revisiting Whistleblower Protection in OECD Countries: From Commitments to Effective Protection. GOV/PGC/ETH(2014)4. [NC]
22. OECD. (2014). Illicit Financial Flows from Developing Countries: Measuring OECD Response. 1-111. [NWH]
23. OECD, UNODC, World Bank. (2013). Anti-Corruption Ethics and Compliance Handbook for Business. <https://www.oecd.org/corruption/Anti-CorruptionEthicsComplianceHandbook.pdf> [NWH]
24. O'Neill, N. (2021). "Recognizing the importance of whistleblowers in healthcare." Nursing **51**(4): 54-56. [NC]
25. Outterson, K. (2012). "Punishing health care fraud--is the GSK settlement sufficient?" N Engl J Med **367**(12): 1082-1085. [NWH]
26. Pomeranz, J. L. and A. R. Schwid (2021). "Governmental actions to address COVID-19 misinformation." Journal of Public Health Policy **42**(2): 201-210. [NWH]
27. PSR Legislative Research. (2010). Legislative Brief. The Public Interest Disclosure and Protection to Persons Making the Disclosures Bill, 2010 (India). [NWH]
28. Rauwolf, P, and Jones, A. (2019). Exploring the utility of internal whistleblowing in healthcare via
29. agent-based models. BMJ Open 2019;9:e021705. doi:10.1136/bmjopen-2018-021705 [NWH]
30. Smith, J. (2005). "Whistleblowers, threats, and bribes: a short history of genetically engineered bovine growth hormone." Genewatch **18**(3): 6-9, 16. [NL]
31. Stafford, R. L. (2017). "Betrayal of local government whistleblowers: Expecting rights and resources, facing harsh realities." International Journal of Human Resources Development and Management **17**(1-2): 162-181. [NWH]
32. Terracol, M. (2021). Are EU Governments Taking Whistleblower Protection Seriously? Berlin: Transparency International. 1-38. [NWH]
33. Thacker, JJ (2021). Assessing the role of whistleblowers. Manuscript posted to ResearchGate. [NC]
34. Transparency International. (2015). Speaking up Safely. Civil Society Guide to Whistleblowing in the Middle East and North Africa Region. Berlin: Transparency International. 1-41. [NWH]
35. UNODC. (2015). Resource Guide on Good Practices in the Protection of Reporting Persons. Vienna: UNODC. <https://www.unodc.org/documents/corruption/Publications/2015/15-04741_Person_Guide_eBook.pdf> [NWH]
36. Zuniga, N. (2020). Gender sensitivity in corruption reporting and whistleblowing. Help Desk Answer 2020:10. U4 Anti-corruption Resource Centre, Norway. <https://www.u4.no/publications/gender-sensitivity-in-corruption-reporting-and-whistleblowing.pdf> [NWH]
